# Origanum majorana L. polyphenols: *in vivo* antiepileptic effect, in silico evaluation of their bioavailability, and interaction with the NMDA receptor

**DOI:** 10.3389/fchem.2023.1257769

**Published:** 2024-01-19

**Authors:** Amal Amaghnouje, Mohamed Chebaibi, Saeed M. Aldossari, Hazem K. Ghneim, Fatima Ez-zahra Amrati, Imane Es-Safi, Francesca Di Cristo, Anna Calarco, Sanae Achour, Fabrizio Carta, Yazeed A. Al-Sheikh, Mourad A. M. Aboul-Soud, Dalila Bousta

**Affiliations:** ^1^ Laboratory of Biotechnology, Health, Agrofood and Environment (LBEAS), Faculty of Sciences Dhar El Mehraz, Sidi Mohamed Ben Abdellah University, Fez, Morocco; ^2^ Ministry of Health and Social Protection, Higher Institute of Nursing Professions and Health Techniques, Fez, Morocco; ^3^ Biomedical and Translational Research Laboratory, Faculty of Medicine and Pharmacy of Fez, Sidi Mohamed Ben Abdellah University, Fez, Morocco; ^4^ Department of Clinical Laboratory Sciences, College of Applied Medical Sciences, King Saud University, Riyadh, Saudi Arabia; ^5^ Laboratory of Cell Biology and Molecular Genetics (LBCGM), Department of Biology, Faculty of Sciences, Ibn Zohr University, Agadir, Morocco; ^6^ National Research Council, Research Institute on Terrestrial Ecosystems (IRET), Porano, Italy; ^7^ NEUROFARBA Department, Sezione di Scienze Farmaceutiche e Nutraceutiche, University of Florence, Florence, Italy

**Keywords:** Origanum majorana L, epilepsy, bioavailability, polyphenolic extract, HPLC, NMDA receptor

## Abstract

**Introduction:** Epilepsy is a chronic brain disease characterized by repeated seizures and caused by excessive glutamate receptor activation. Many plants are traditionally used in the treatment of this disease. This study aimed to evaluate the bioavailability of a polyphenolic extract obtained from *Origanum majorana* L. (OMP) leaves, as well as its antiepileptic activity and its potential mechanism of action.

**Methods:** We have developed and validated a simple, rapid, and accurate stability-indicating reversed-phase liquid chromatographic method for the simultaneous determination of caffeine and quercetin in rat plasma. The OMP antiepileptic effect was evaluated with pilocarpine-induced seizures, and a docking method was used to determine the possible interaction between caffeic acid and quercetin with the N-methyl-D-aspartate (NMDA) receptor.

**Results and Discussion:** Both compounds tested showed low bioavailability in unchanged form. However, the tested extract showed an anticonvulsant effect due to the considerably delayed onset of seizures in the pilocarpine model at a dose of 100 mg/kg. The molecular docking proved a high-affinity interaction between the caffeic acid and quercetin with the NMDA receptor. Taken together, OLP polyphenols demonstrated good antiepileptic activity, probably due to the interaction of quercetin, caffeic acid, or their metabolites with the NMDA receptor.

## 1 Introduction

Approximately 50 million individuals worldwide are believed to be affected by epilepsy. Up to 80% of these cases originate from less developed countries (Devinsky et al., 2018). Approximately 70 percent of individuals diagnosed with epilepsy could be seizure-free if they received timely and appropriate treatment. Approximately three-fourths of people living in low-income countries who are suffering from epilepsy are not afforded access to proper care. In certain nations, the number of individuals with epilepsy who do not obtain suitable treatment reaches ninety percent. In these countries, the lack of proper training among healthcare practitioners leads to difficulty in adequately recognizing, diagnosing, and treating individuals suffering from epilepsy. In most Low-income countries, anti-epileptic drugs are not available (Milligan, 2021). Therefore, several plants are traditionally utilized as complementary and alternative medicine including *Origanum majorana* L. (*O. majorana).*

Polyphenols are phytochemical compounds of plant-origin that are characterized by powerful antioxidant properties. Previous studies have shown that polyphenols have been tested as a treatment for several neurological disorders, including epilepsy. Quercetin, epigallocatechin gallate, and resveratrol, have been demonstrated to have the ability to mediate various signaling processes involved in epilepsy (Dhir, 2020). Other reports showed that lycopene and curcumin possess a significant anti-epileptic effect via eliminating reactive oxygen species (Yang et al., 2020). Concerning the present work, there are no studies are available about the antiepileptic effect of polyphenols from *Origanum majorana* L. polyphenol has never cited.

According to our previous phytochemical report, quercetin and caffeic acid (CA) are the main effective polyphenolic compounds of marjoram (Amaghnouje et al., 2020), which were found to have various activities, such as anticancer, antioxidant, anti-inflammatory, antimicrobial, and antiepileptic properties (Far et al., 2017).

To accomplish rational clinical application and optimal drug dosage, the study of bioavailability is necessary. Moreover, the dose proportionality of polyphenols treatment is important for its safety and efficacy, as it may indicate whether the dose-response relationship is linear or not, which can affect efficacy and toxicity.

In this study, the concentrations of caffeic acid and quercetin in the plasma of rats was determined by use of HPLC-UV after oral administration. Next, the antiepileptic effect of the polyphenols derived from *O. majo*rana L. was evaluated in a pilocarpine model. Finally, an *in silico* approach utilizing molecular docking was conducted to investigate the potential mechanism-of-action (MOA) of the extract by use of receptor-ligand analysis.

## 2 Results

### 2.1 Bioavailability study

A set of diverse experiments were conducted to optimize the mobile phase. This optimization aims to obtain symmetrical peaks and an optimal runtime for analyzing caffeic acid and quercetin. Reversed-phase high-performance liquid chromatography (RP-HPLC) is a commonly employed method for separating pharmaceutical compounds. Initially, a C18 column and polar solvents were used in the study. Adjustments were made to the mobile phase composition, column flow rate, and temperature to identify the most effective chromatographic conditions that ensured efficient separation and minimized the analysis time.

**FIGURE 1:**
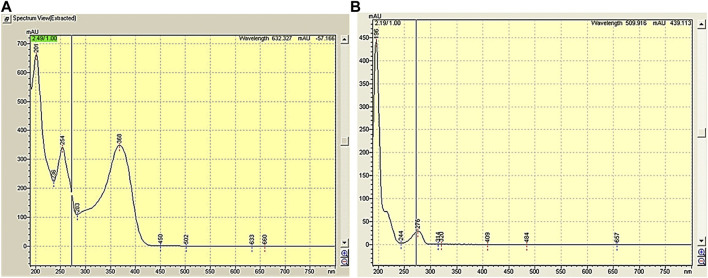
**(A)** Caffeic acid spectrum, **(B)** Quercetin spectrum.

A mobile phase comprising a mixture of acetonitrile and water (55:45) pH 3.12 was utilized to elute caffeic acid and quercetin. As shown in Figure 1, quercetin was detected at 272 nm, whereas caffeic acid was detected at 320 nm. Acetonitrile was advantageous due to its minimal interference at shorter wavelengths. By changing the wavelength, improvements were observed in terms of absorbance and the calculated number of plates, despite the persistence of peak fronting. Hence, pH was adjusted to 3.12 using orthophosphoric acid (OPA) to address this concern. Eventually, this composition and pH proved successful in resolving this issue.

### 2.2 Method validation

#### 2.2.1 Linearity

**FIGURE 2:**
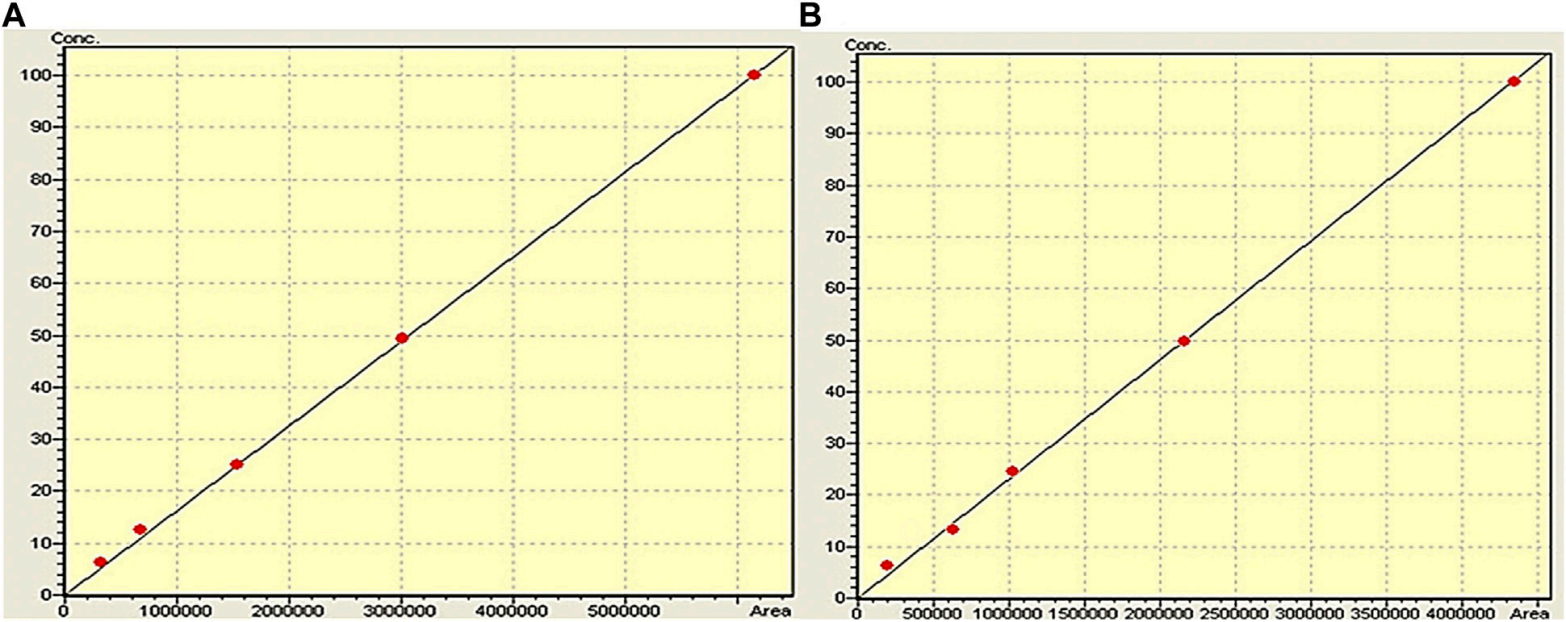
Calibration curve of caffeic acid **(A)** and quercetin **(B)**.

A range of dilutions were prepared across different concentration levels to establish linearity. The two polyphenols were tested within a concentration range of 100–6.25 μg/mL. Triplicates were prepared for each concentration and subjected to analysis. The employed standard concentrations of 100, 50, 25, 12.5, and 6.25 μg/mL demonstrated perfect linearity. A linear regression was obtained where peak areas were plotted against their corresponding concentrations **(** Figure 2). The resultant calibration curve was utilized to derive linear regression equations and calculate correlation coefficients. For quercetin, the linear equation was determined as Y = 0.959x (*R* ^2^ = 0.999), while for caffeic acid, the linear equation was Y = 0.976x (*R* ^2^ = 0.999). Y represents the dependent variable in these equations, and x represents the independent variable. The slopes, 0.959 and 0.976, indicate the rate of change in the dependent variable (Y) per unit change in the independent variable (x). It should be noted that the *y*-intercept was set to 0, indicating that the value of the variable Y is 0 when x = 0. The concentrations within the specified range demonstrated excellent linearity **(** Figure 2).

#### 2.2.2 Limit of detection (LOD) and lower limit of quantification (LOQ)

To determine the LOD and LOQ dilutions series of the two polyphenols under investigation were prepared. The calculation of LOD and LOQ was based on standard deviation (SD) of the response and the slope of the standard curve. SD of the analyte was determined based on the SD of the blank. An adequate number of samples near the detection limit (DL) was included to ensure method validation.

At a signal-to-noise (S/N) ratio of 3:1, the DL for caffeic acid and quercetin were determined to be 0.008 μg/mL and 0.007 μg/mL, respectively. For the calculation of LOQ, S/N ratio of 10:1 was employed, resulting in values of 0.022 μg/mL for quercetin and 0.021 μg/mL for caffeic acid (Table 1).

**TABLE 1:** LOD and LOQ for the analysis of caffeic acid and quercetin, in standard solutions.

Molecule	Calibration curve	Correlation coefficient (r)	LOD (μg/mL)	LOQ (μg/mL)
**Caffeic acid**	Y = 0.976x	0.999	0.008	0.021
**Quercetin**	Y = 0.959x	0.999	0.007	0.022

Molecule of caffeic acid and quercetin.

#### 2.2.3 Precision and accuracy

To assess the accuracy of this approach, specific internal standards were introduced into the sample solutions. Each concentration range was examined three times to ensure consistency. The obtained results exhibited a high level of precision when compared to the actual values. The recovery rates were determined to be within the range of 94%–99%. To evaluate both inter-day and intra-day precision, two different concentrations were selected and employed in the recovery investigations. The accuracy was expressed as a relative standard deviation (RSD) percentage. For the inter-day precision, involving nine repetitions for each concentration, the RSD values ranged from 0.08% to 0.09% for quercetin and 0.29%–0.30% for caffeic acid. As for the intra-day precision, conducted with six repetitions, the RSD values ranged from 0.25% to 0.3% for quercetin and 0.18%–0.2% for caffeic acid (Table 2). The recovery percentage obtained after the study was deemed satisfactory. These results clearly confirmed the precision of the developed method.

**TABLE 2:** Accuracy of the developed HPLC method.

Standard	Concentration µg/mL	Intraday			Interday			
		Recovery concentration, μg/mL	Accuracy^a^, %	Recovery, %	Recovery concentration, μg/mL	Accuracy^b^, %	Recovery, %	
**Quercetin**	50	46.51	0.08	93	47.35	0.25	94	
	25	24.28	0.09	97	24.33	0.3	97	
**Caffeic acid**	50	47.00	0.29	94	49.42	0.18	98	
	25	25.4	0.30	101	24.87	0.2	99	

^a^ Expressed in RSD (%), 9 repeated measurements.

^b^ Expressed in RSD (%), 6 repeated measurements.

Standard in HPLC.

#### 2.2.4 Bioavailability study

**FIGURE 3:**
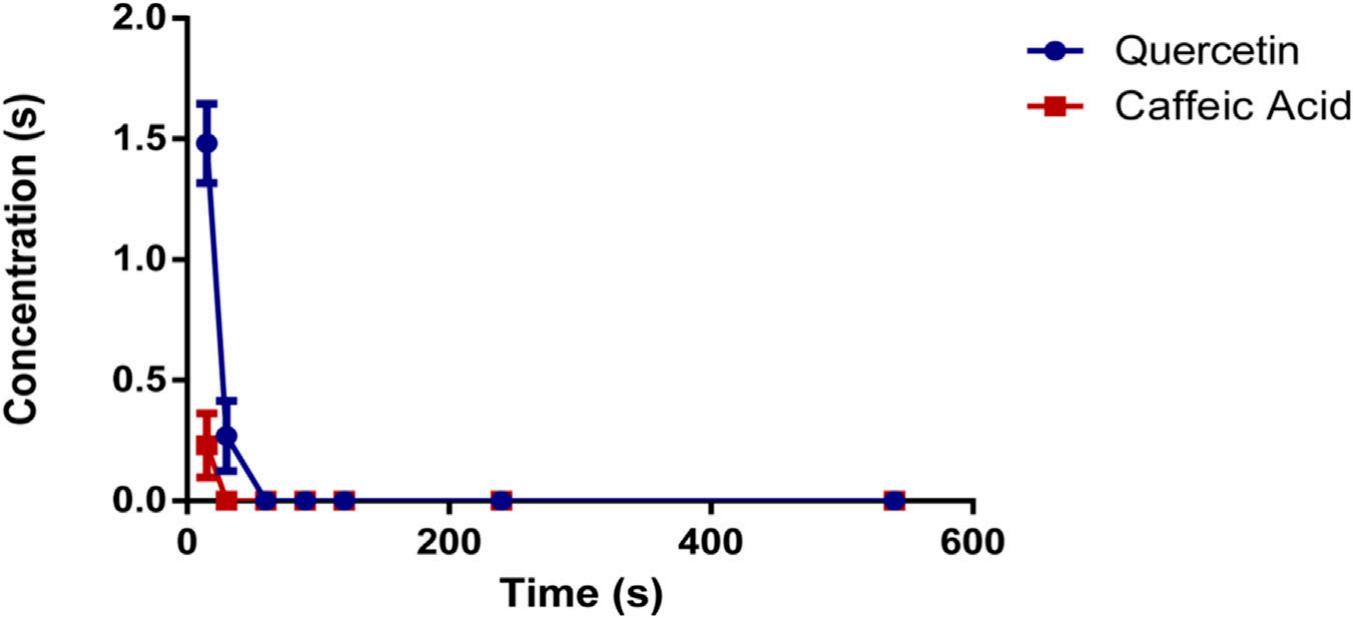
Plasma concentration of quercetin and caffeic acid following the oral administration of polyphenols derived from *Origanum majorana* L.

The method described was applied to measure the plasma concentrations of quercetin and caffeic acid in rat’s blood that had been orally administered O. majorana L. derived polyphenols (OMP) at a dose of 100 mg/kg. The mean plasma concentration of OMP against the time post-administration of the analytes is represented in Figure 3. Quercetin was detected 15 min after oral administration with an average of 1.481 μg/mL, and then decreased after 30 min to 0.268 μg/mL. whereas caffeic acid was only detected 15 min post-administration of OMP at a dose of 0.230 μg/mL.

### 2.3 Antiepileptic effect of OMP

The antiepileptic properties of OMP were investigated by examining their impact on seizures induced by pilocarpine injection in mice. The observation of the behavior of the animals after the injection of pilocarpine indicated the development of immobility, contractions of the ear and contractions of the vibrissae, closing of the eyes, salivation, facial clonus, episodic clonus of the limbs, and nodding of the head (stages 1–3 on the Racine scale).

#### 2.3.1 Status epilepticus

Status induction was significantly decreased by 40% when mice were pretreated with a high dose of polyphenols, OMP (100 mg/kg), which is comparable to that of sodium valproate (a standard anti-epileptic drug) at 300 mg/kg (Table 3).

**TABLE 3:** Percentage induction of status epilepticus.

	Pilo (360 mg/kg)<	OMP		Sodium valproate 300 mg/kg
		50 mg/kg	100 mg/kg	
**SE (%)**	100	60	40	40

Status epilepticus percent.

#### 2.3.2 Tonic-clonic seizures

**FIGURE 4:**
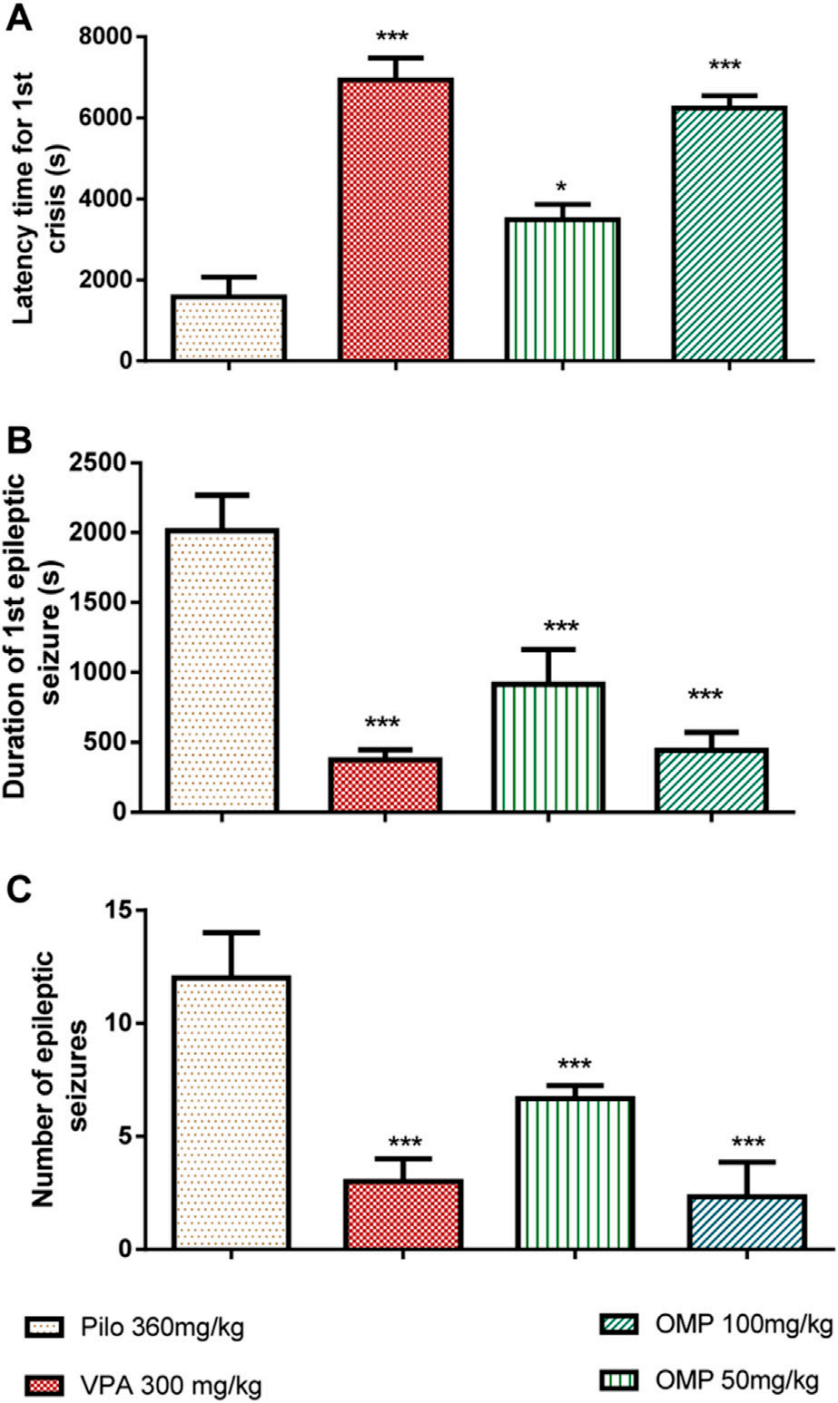
The impact of OMP on pilocarpine-induced status epilepticus (PILO) was evaluated. The following parameters were measured: **(A)** Latency time for the first seizure, **(B)** Duration of the first seizure, and **(C)** Number of epileptic seizures. The results were presented as the mean ± standard error of the mean (SEM) with a sample size of 10 (n = 10).

Results indicated that OMP treatment resulted in a dose-dependent delay of the onset of clonic (*p* < 0.001) and tonic (*p* < 0.001) seizures (Figure 4).

The polyphenols (100 mg/kg) showed comparable effects to sodium valproate, the standard anti-epileptic agent (300 mg/kg), in increasing latencies to clonic and tonic seizure. Obtained data showed that OMP is less potent than the positive control in reducing seizure duration and delaying seizure onset, but their efficacy was comparable. The administration of an oral dose of OMP at 100 mg/kg exhibited a significant anticonvulsant effect, effectively safeguarding animals from mortality resulting from pilocarpine-induced convulsions (Table 4).

**TABLE 4:** Percentage of protection of OMP in mice after pilocarpine injection.

	Pilo (360 mg/kg) (%)	OMP		Valproate 300 mg/kg
		50 mg/kg	100 mg/kg	
**Survival percentage**	0	62.5% ***	70% ***	80% ***

Percentage of protection of OMP in mice after pilocarpine injection.

### 2.4 *In silico* study

N-methyl-D-aspartate receptors (NMDARs) are the ionotropic receptor type that has a significant role in the pathophysiology of various neurological disorders, such as Alzheimer’s disease (Cacabelos et al., 1999), epilepsy, Parkinson’s and Huntington’s diseases (Beal, 2004; Fan and Raymond, 2007). The excessive influx of calcium (Ca^2+^) resulting from subsequent overstimulation of NMDARs directly contributes to the pathophysiology of these neurological disorders (Sattler and Tymianski, 2000). The activation (open state) of NMDARs depends on the binding of a glutamate agonist and a co-agonist such as _D_-Serine or Glycine. NMDARs are characterized by slow kinetics and permeability to ions such as Na^+^, K^+^, and Ca^2+^ (Dingledine et al., 1999; Cull-Candy et al., 2001). Blocking NMDAR activity is a key step in the treatment of many neurological diseases, including epilepsy.

To verify the inhibitory effect of quercetin and caffeic acid derived from O. majorana polyphenols on human NMDA receptors, human GluN1-GluN2A and GluN1-GluN2B NMDA receptors were chosen for this *in silico* study. During our molecular docking experiments, quercetin and caffeic acid exhibited an inhibitory effect in the active sites of GluN1-GluN2A and GluN1-GluN2B receptors. The binding free energy (glide score) for quercetin was −6.374 kcal/mol in the active site of GluN1-GluN2A and −7.061 kcal/mol in the active site of GluN1-GluN2B. For caffeic acid, the binding free energy was −5.633 kcal/mol in the active site of GluN1-GluN2A and −5.641 kcal/mol in the active site of GluN1-GluN2B (Table 5).

**TABLE 5:** Docking results with quercetin and caffeic acid in the human GluN1–GluN2A and GluN1–GluN2B NMDA receptors (PDB: 7EU7 and PDB: 7EU8).

Compound name	Compound ID	Formula	Receptor	Glide score (kcal/mol)	Glide energy (kcal/mol)	Glide emodel (kcal/mol)
**Quercetin**	5280343	C_15_H_10_O_7_	7EU7	−6.374	−38.926	−52.219
**Caffeic acid**	689043	C_9_H_8_O_4_		−5.633	−33.313	−43.259
**Quercetin**	5280343	C_15_H_10_O_7_	7EU8	−7.061	−42.66	−58.455
**Caffeic acid**	689043	C_9_H_8_O_4_		−5.641	−28.4	−35.791

Molecule of caffeic acid and quercetin.

**FIGURE 5:**
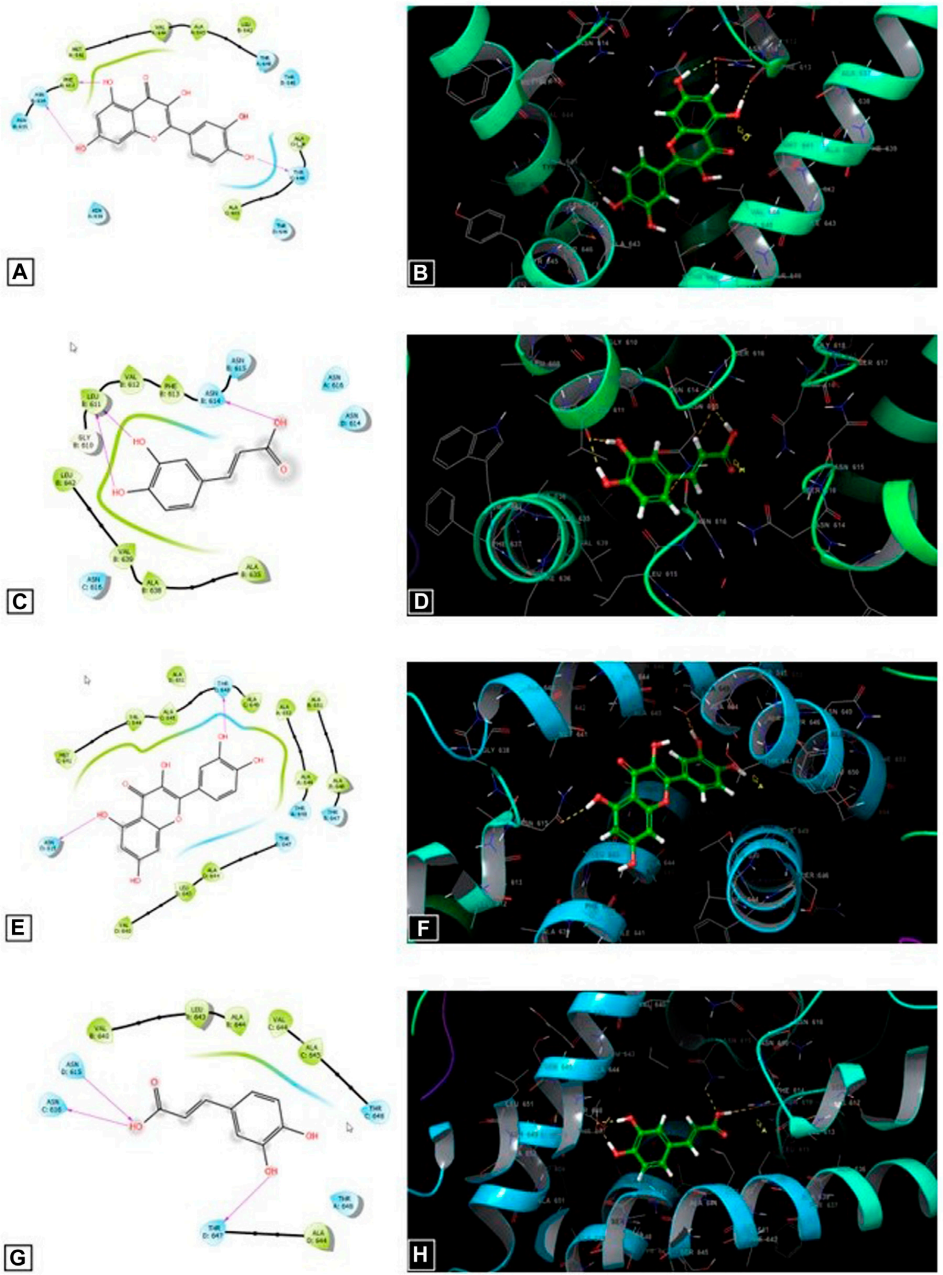
The 2D **(A)** and 3D **(B)** diagrams of the quercetin interactions in the active side of the GluN1–GluN2A NMDA receptors (PDB: 7EU7). The 2D **(C)** and 3D **(D)** diagrams of the caffeic acid interactions in the active side of the GluN1–GluN2A NMDA receptors (PDB: 7EU7). The 2D **(E)** and 3D **(F)** diagrams of the quercetin interactions in the active side of the GluN1–GluN2B NMDA receptors (PDB: 7EU8). The 2D **(G)** and 3D **(H)** diagrams of the caffeic acid interactions in the active side of the GluN1–GluN2A NMDB receptors (PDB: 7EU8).

Figure 5 shows that quercetin contributes to the formation of three hydrogen bonds with residues PHE 613, ASN 614, and THR 648 in the active sites of the GluN1- GluN2A NMDA receptors. In addition, it forms two hydrogen bonds with residues ASN 615 and THR 648 in the active site of GluN1—GluN2B NMDA receptors. By contrast, caffeic acid contributes to the formation of three hydrogen bonds with residues LEU 611 and ASN 614 in the active sites of the GluN1- GluN2A NMDA receptors. Furthermore, it forms 3 hydrogen bonds with residues ASN 615, ASN 616, and THR 647 in the active site of GluN1—GluN2B NMDA receptors.

### 2.5 ADME prediction

The parameters examined to predict the ADME (absorption, distribution, metabolism, and excretion) of quercetin and caffeic acid yielded acceptable results. Both compounds exhibited a molecular weight below 500, and their central nervous system (CNS) activity parameter fell within the range of −2 to +2. In this range, a value of −2 indicates inactivity, whereas a value of +2 signifies activity (Table 6). The calculated partition coefficients between blood and brain indicate favorable values of −2.074 for quercetin and −1.010 for caffeic acid. Moreover, the predicted human oral absorption percentages are 61.013% for quercetin and 63.614% for caffeic acid (Table 6).

**TABLE 6:** ADME properties of quercetin and caffeic acid.

Compound name	MW^a^	CNS^b^	PSA^c^	SASA^d^	QPlogPo/w^e^	QPlogBB^f^	QplogS^g^	% Human oral absorption^h^
Quercetin	302.240	−2	91.897	455.795	6.934	−2.074	−3.378	61.013
Caffeic acid	180.160	−2	72.375	301.155	−0.667	−1.010	−1.010	63.614

^a^ The molecular mass falls within 500 atomic mass units.

^b^ The impact on the central nervous system ranges from −2, to +2.

^c^ The polar surface area is within the range of 7.0–200.

^d^ The solvent-accessible surface area, determined using a probe with a radius of 1.4, falls within the range of 300–1000 radius units.

^e^ The predicted partition coefficient between octanol and waterfalls within the range of −2, to 6.5.

^f^ The predicted partition coefficient between blood and brain falls within −3, to 1.2.

^g^ The predicted aqueous solubility, represented as S in mol/dm³, falls within the range of −6.5 to 0.5.

^h^ The predicted human oral absorption is expressed on a 0%–100% scale, where <25% indicates poor absorption and >80% indicates high absorption.

## 3 Discussion

Quercetin and caffeic acid are the primary active constituents present in the polyphenols of *O. majorana*, known for their notable anxiolytic and antidepressant effects. However, there is limited available data on the bioavailability characterization of quercetin and caffeic acid in rats following the oral administration of *O. majorana* polyphenols. This study presents the first report on the bioavailability assessment of polyphenols extracted from O. *majorana* L. using HPLC-UV. The findings revealed that quercetin and caffeic acid were rapidly absorbed and metabolized within 30 min after oral administration. Multiple studies, conducted in animal models or on human subjects, have provided evidence supporting the rapid absorption and metabolism of quercetin and caffeic acid (Chen et al., 2005; Mullen et al., 2006; Lafay and Gil-Izquierdo, 2008; Moon et al., 2008; Dabeek and Marra, 2019; Kishida and Matsumoto, 2019).

Quercetin belongs to the flavonoid class, specifically the flavanol subgroup. In its unchanged form, it exhibits low bioavailability and aqueous solubility, but the conjugated form demonstrates high bioavailability (Batiha et al., 2020). In the research conducted by Chen et al., it was observed that only 5.3% of quercetin in its unchanged form exhibited bioavailability, while the total absorbed quercetin accounted for 59.1%. Following oral administration, approximately 93.3% of quercetin was metabolized in the gut, with a mere 3.1% undergoing hepatic metabolism (Chen et al., 2005).

Mullen et al. investigated the presence of quercetin metabolites in blood and urine following the consumption of quercetin-rich food products. The identified metabolites included quercetin-3, 4′-O-diglucoside, quercetin-3-O-glucoside, quercetin-4′-O-glucoside, isorhamnetin-4′-O-glucoside, and quercetin-3-O-rutinoside (Mullen et al., 2006). Quercetin exhibits a short half-life and is quickly eliminated from the bloodstream. Its metabolites were detected in plasma half an hour after intake, but substantial quantities were excreted within a 24-h timeframe (Moon et al., 2008; Batiha et al., 2020). In a study conducted by Yang et al., in 2022, it was observed that upon oral ingestion of quercetin, quercetin glucuronides and sulfates were detected shortly after administration (within 5 min) and remained confined to the bloodstream. This finding suggests a remarkably swift absorption of quercetin and simultaneous sulfation and glucuronidation during the absorption process. During the elimination phase, supplementary peaks of quercetin conjugates were identified, suggesting the presence of enterohepatic circulation for these metabolites. However, no unmodified quercetin forms were detected, indicating that the systemic bioavailabilities of quercetin were essentially negligible (Yang et al., 2005). *In vitro* experiments conducted by Carbonaro and Grant demonstrated that approximately 1.5% of quercetin penetrated the intestinal wall, with over half of the total quercetin being bound to the tissue of the small intestine (Carbonaro and Grant, 2005). Numerous investigations focusing on caffeic acid have reported its rapid uptake by the stomach or small intestine, as well as its uptake by detoxification enzymes in the intestinal and/or hepatic regions (Lafay and Gil-Izquierdo, 2008; Kishida and Matsumoto, 2019).

In a study conducted by Azuma et al., it was demonstrated that rats administered with caffeic acid via gastric intubation at a dosage of 700 μmol/kg exhibited the presence of this compound, along with ferulic acid, and their principal metabolites were identified as sulfates or glucuronides. These metabolites were detected in plasma within 30 min after intubation, and their concentrations reached a peak approximately 2 h later. Nevertheless, whatever the dose administered, most of the conjugated caffeic and ferulic acids disappeared after 6 h (Azuma et al., 2000; Lafay and Gil-Izquierdo, 2008). After oral caffeic acid administration to rats, the compound reached its highest concentration within 10 min. Specifically, it was found to have a maximum concentration of 11.2 µM in the portal vein and 2.3 µM in the aorta (Konishi et al., 2005). An investigation demonstrated the stomach’s capability to absorb caffeic acid following gastric administration of 2.5 µM. Subsequently, plasma analysis revealed the presence of 0.36 µM of unconjugated caffeic acid and 0.51 µM of the conjugated form in the aorta after a 5-min infusion period (Konishi et al., 2005). Other work has shown that around 19.1% of caffeic acid can be absorbed from the tiny intestine (Lafay and Gil-Izquierdo, 2008).

In our study, we utilized the pilocarpine mouse model to investigate the *in vivo* effects of antiepileptic compounds. Pilocarpine, a highly potent muscarinic agonist, was administered to induce notable behavioral alterations. This model serves as a valuable tool for exploring the mechanisms underlying human temporal lobe epilepsy and assessing the efficacy of potential anticonvulsant medications (Scorza et al., 2009).

The findings presented in this report propose a potential mechanism-of-action for the observed anticonvulsant effects of marjoram leaves, highlighting the involvement of polyphenols such as flavonoids and phenolic acids. These compounds may contribute to their antiepileptic properties through mechanisms such as antioxidant activity. Moreover, existing literature extensively documents the ability of flavonoids and phenolic acids to enhance GABA neurotransmission. GABA, the primary inhibitory neurotransmitter, is crucial in mitigating epilepsy seizures (Johnston, 2015; Rebas et al., 2020). Consequently, the dose-dependent suppression of seizures by OMP (marjoram extract) may be attributed to its phenolic acid composition. This composition potentially enhances GABA neurotransmission in the brain or inhibits glutamate neurotransmission mediated by NMDA receptors.

The antiepileptic activity of *O. majorana* could be explained by molecular docking results **(** Table 5; Figure 5)**.** Different substances are used by the central nervous system as neurotransmitters. Glutamate is the main neurotransmitter for both excitatory and inhibitory action. About 30 000 synapses, 95% of which are excitatory, are found on cortical pyramidal neurons, the glutamate is the main excitatory neurotransmitter in the brain. Among glutamate receptors, NMDA have been considered the most well investigated receptor (Hanada, 2020). NMDA receptors (NMDARs) have been reported to be involved in neurodegenerative illnesses such as epilepsy (Aseervatham et al., 2016). Caffeic acid was found to have neuroprotective effects against DNA damage and oxidative stress caused in the kindling epilepsy model. Additionally, it has been reported to reduce ROS levels produced after pentylenotetrazole inducing seizures in mice (Coelho et al., 2015).

Blockade of NMDARs decreases brain damage related to epilepsy. NMDARs are considered druggable targets for antiepileptic therapeutics. NMDAR agonists can cause epileptic seizures in humans as well as animal models, while antagonists inhibit those seizures (Celli and Fornai, 2021). In the same context as NMDAR antagonists, the antiepileptic effect of O. majorana polyphenols may be due to the action of its phenolic compounds, especially quercetin and caffeic acid, which demonstrated interesting glide scores in inhibiting NMDARs.

On the other hand, quercetin has been reported to possess multiple pharmacological activities, including anti-inflammatory and neuroprotective activity. It is documented that quercetin enhance the expression of NMDAR, which may explain its potential to improve memory and synaptic plasticity to a seizure (Moghbelinejad et al., 2016). Other studies demonstrated that quercetin pretreatment prevented the decline in NMDAR expression (Tongjaroenbuangam et al., 2011). Additionally, it has been discovered that quercetin protects against a variety of neurological diseases, including epilepsy. Quercetin has been shown to have antiepileptic effects in animal models, via reducing oxidative damage and the neuroinflammation, involved in the epileptic processes (Akyuz et al., 2021). Reactive oxygen species (ROS play an advantageous role as “redox messengers” in the balance between oxidants and antioxidants. However, ROS excess can lead to epileptogenesis. So, the obtained effect can be explained by the capacity of the phenolic compounds present in the tested extract to inhibit ROS and consequently inhibit the deleterious effect of oxidative stress (Borowicz-Reutt and Czuczwar, 2020).

The compounds were designed with lipophilicity as a crucial factor for their activity on the central nervous system (CNS). The predicted values for QPlogBB and CNS activity demonstrate that the selected compounds exhibit CNS activity and have the potential to cross the blood-brain barrier (BBB). Typically, compounds intended for the treatment of central nervous system (CNS) disorders such as epilepsy should have a reduced polar surface area (PSA). In this study, the predicted PSA values for the selected compounds fell within a range of 72.375–91.897. These results indicate that the chosen compounds possess a lower polar surface area, suggesting their potential to traverse the blood-brain barrier (BBB).

The cholinergic system of the brain modulates neuronal excitability and synaptic transmission. According to the results of several previous studies, the antiepileptic effect of our polyphenolic extract of *O. majorana* could be due to the interaction of OMP with the cholinergic system (Büget et al., 2016; Hajlaoui et al., 2016). In other studies, Akünal Türel et al. found that *Origanum majorana* L. ethanolic extract presented a high level of rosmarinic acid, known for its antioxidant effect in protecting brain microglial cells against death induced by oxidative stress (DZHAFAR et al., 2020; Akünal Türel and Yunusoğlu, 2023).

## 4 Conclusion

The present study explored the potential *Origanum majorana* L. polyphenols (OMP) as a treatment for epilepsy via assessing the bioavailability of the tested polyphenolic extract and examining its antiepileptic activity, with a focus on the interaction of its compounds with the N-methyl-D-aspartate (NMDA) receptor. Results revealed that both caffeine and quercetin from the OMP extract exhibited low bioavailability in their unchanged forms. However, the extract demonstrated a significant anticonvulsant effect by delaying the onset of seizures in a pilocarpine-induced seizure model, particularly at a dose of 100 mg/kg. Molecular docking analysis further supported these observations, indicating a high-affinity interaction of caffeic acid and quercetin with the NMDAR. The OMP polyphenols, specifically quercetin and caffeic acid, or their metabolites, displayed notable antiepileptic activity, likely attributed to their interaction with the NMDAR. These findings provide valuable information on the therapeutic benefits of OMP polyphenols in the management of epilepsy, thanks to their phenolic compounds: quercetin and ferulic acid. This could constitute a promising therapeutic natural avenue to control epileptic seizures.

## 5 Materials and methods

### 5.1 Plant

Marjoram (*Origanum majorana* L.) leaves were acquired from Rissani city, Southern Morocco. Identification and authentication were conducted by Professor A. Bari, who is a Botanist at the Faculty of Science Dhar El Mahraz Fez). Plant specimen was assigned voucher number DACB: BPRN74, LBEAS laboratory.

### 5.2 Preparation of polyphenolic extract

Methanol (150 mL) was used to extract 50 g of *O. majorana* powder. Following the concentration of the solvent, the resulting extract was subsequently dissolved in 250 mL of distilled water. The aqueous extract underwent extraction with 100 mL of hexane, chloroform, and ethyl acetate solvents. The ethyl acetate phase underwent concentration utilizing a BUCHI 461 rotary evaporator. The resultant residue comprised the polyphenolic extract (Amrati et al., 2023).

### 5.3 Animals

While male Swiss mice, weighing 25–35 g, were purchased from the Pasteur Institute in Rabat (Morocco), Wistar rats were obtained from the Emirates Wildlife Propagation Center (ECWP) in Missour (Morocco). Animals were housed in cages with 20 mice or 5 rats per cage, maintaining a room temperature of 23°C ± 2°C. They had *ad libitum* access to water and food and were subjected to a 12:12 h light/dark cycle. All experimental manipulations were conducted between 8:00 a.m. and 3:00 p.m. These experiments were conducted according to the ethical principles of animal experimentation ensuring the implementation of all the necessary biosecurity measures; whereby maintaining optimal experimental conditions and safeguard animal safety. The surgical equipment and storage devices in the operating room were autoclaved before each use.

### 5.4 Bioavailability study

#### 5.4.1 Chemicals and reagents

Quercetin and Caffeic acid (CA)) were obtained from Sigma (St. Louis, MO, United States of America). Analytical grade acetonitrile and orthophosphoric acid (OPA) were utilized, while double distilled water was prepared by employing a micropore nylon filter with a pore size of 0.45 µm.

#### 5.4.2 Analytical equipment

The HPLC system employed in this study is equipped with a quaternary pump type “LC-20”, an automatic injector “DIL-20”. The outlet of the column is connected to UV-Visible detector with diode arrays “SPD-M20A”. Data were processed by use of the LC solution software. For the HPLC analyses, the mobile phase consisted of a mixture of acetonitrile and water in a ratio of 55:45 (v/v). Chromatographic separation was conducted at 25°C with an injection volume of 20 μL. The pH of the mobile phase was adjusted to 3.12 by use of orthophosphoric acid, and a flow rate of 1 mL/min was maintained. The detection was set at two wavelengths, namely, 210 and 254 nm.

#### 5.4.3 Standard solutions preparation

One milligram of every standard was dissolved in 10 mL of acetonitrile/water solution (mobile phase) and subsequently diluted to obtain the following serial dilutions 100, 50, 25, 12.5, and 6.25 ppm.

#### 5.4.4 Method validation

Validation of this method was conducted in accordance with the International Council for Harmonization of Technical Requirements for Pharmaceuticals for Human Use (ICH) guidelines. The performance characteristics of the method were established including accuracy, specificity, precision, and the limits of quantification and detection.

##### 5.4.4.1 Linearity

Linearity was established by creating a minimum of five calibration curves, where the ratio of peak area for the standard analytes were plotted against their respective concentrations. For calibration curves to be considered valid, correlation coefficient values of ≥0.99 were required. The linearity range for each standard was 100, 50, 25, 12.5, 6.25 μg/mL. Each standard concentration was studied three times. The *x*-axis of the graph was employed to plot all concentrations, whereas the *y*-axis was utilized to plot the corresponding peak areas. The linearity of the calibration curve was evaluated through linear regression analysis.

##### 5.4.4.2 Precision and accuracy

To assess precision and accuracy, the relative standard deviation (RSD) was determined for intra-day and inter-day analyses. For the evaluation of intra-day precision, three standards were employed for recovery studies and injected in six replicates on the same day. Nine replicates of the same standard were tested over three consecutive days to evaluate consistent accuracy. All samples were assessed in triplicates.

##### 5.4.4.3 Lower Limit of quantification and Limit of detection

The **Lower Limit of Quantification** (LOQ) was the lowest concentration on the calibration curve with acceptable precision (RSD) not surpassing 20% and relative error (RE) of accuracy must not deviate by more than 20%. The **Limit of Detection** LOD was the lowest concentration that can be perceived with statistical significance using a given analytical procedure.

#### 5.4.5 Bioavailability study

This validated method was used to analyze plasma concentrations of AC and Q in rats after an oral administration of polyphenols from *O. majorana* L. (100 mg/kg). Rats were fasted for the first 4 h with access to water after oral administration. Heparinized tubes were used to collect blood samples from the tail vein at various intervals following oral administration, precisely at 15, 30, 60, 120, 240, and 540 min. The plasma was centrifuged (5000 rpm; 10 min). The collected plasma was kept in Eppendorf tubes at −20°C for analysis.

### 5.5 Antiepileptic study

Pilocarpine was injected *via* the intraperitoneal route at a dose of 360 mg/kg to generate the mouse seizure model, following the OMP oral administration, at 50 and 100 mg/kg. Epiliptic seizure behavior of each mouse was observed for 180 min, and ranked in accordance with the modified version of the Racine scale (Racine, 1972). Throughout the six stages, various parameters were observed:

**Stage 1**: Behaviors such as immobility, closed eyes, ear twitching, vibration twitching, sniffling, and facial clonus were detected.
**Stage 2:** Head nodding was observed in conjunction with increased facial clonus.
**Stage 3:** Clonus specifically manifested in the forelimb.
**Stage 4:** Bilateral clonus occurred along with straightening, without any instances of falling.
**Stage 5:** Generalized tonic-clonic seizures occurred, with or without accompanying standing up and falling. Parameters recorded for this stage included the latency time of the first tonic-clonic seizure, the duration of the first seizure, the total number of seizures, and the presence of an epileptic state.

Finally, the survival percentage of mice within each experimental group was determined after 24 h.

### 5.6 *In silico* study

#### 5.6.1 Preparation of ligands

Structures of quercetin and caffeic acid were acquired from PubChem in SDF format, while the ligands were prepared for docking calculations utilizing the LigPrep tool within the Schrödinger Software program (version 11.5). The ligands were prepared with the OPLS3 force field, and considering ionization states (pH 7.0 ± 2.0), 32 stereoisomers were generated for each compound (Aboul-Soud et al., 2022).

#### 5.6.2 Proteins preparation

The PDB format of the human GluN1-GluN2A (PDB: 7EU7) and GluN1-GluN2B (PDB: 7EU8) NMDA receptor crystal structures in three dimensions were acquired from the protein data bank (Zhang et al., 2021; Ladagu et al., 2023). The structures were refined and prepared using the Protein Preparation Wizard in the Schrödinger-Maestro program (v11.5). Modifications included the removal of water molecules, bonding hydrogens to heavy atoms, the substitution of methionines with selenomethionines, and the subsequent assignment of charges and bond orders. Minimization was performed using the OPLS3 force field, resulting in a maximum heavy atom RMSD of 0.30 Å (Aboul-Soud et al., 2022).

#### 5.6.3 Receptor grid generation

A ligand atom was selected to create a default grid box with a volumetric spacing 20 × 20 × 20. The ligand was then connected to the protein-generated grid box using the ‘Extra Precision’ (XP) method. The results were evaluated using the XP GScore.

#### 5.6.4 Glide standard precision (SP) ligand docking

The Glide module in Schrödinger-Maestro (v11.5) was utilized to perform flexible ligand docking with standard precision (SP). Penalties were applied for non-cis/trans amide bonds, and the van der Waals scaling factor and partial charge cutoff parameters for ligand atoms were set to 0.80 and 0.15, respectively. Energy minimization was conducted on the poses, and the final scoring was performed using the Glide score. The best-docked pose with the lowest Glide score value was identified and recorded for each ligand (Aseervatham et al., 2016).

### 5.7 ADME prediction

The absorption, metabolism, distribution, and excretion properties of quercetin and caffeic acid were assessed using the Qikprop feature available in the Schrödinger Software’s Maestro 11.5 version. Predictions were made by considering various physicochemical characteristics and pharmacokinetic properties, including molecular weight, Central nervous system, polar surface area, total solvent surface area, the blood-brain partition coefficient, the octanol/water partition coefficient, as well as aqueous solubility (Chebbac et al., 2023).

## Data Availability

The original contributions presented in the study are included in the article/Supplementary material, further inquiries can be directed to the corresponding authors.

## Abbreviations

CA, caffeic acid; HPLC, high performance liquid chromatography; LOD, limit of detection; LOQ, lower limit of quantification; NAMDR, N-methyl-D-aspartate receptors; NMDA, N-methyl-D-aspartate; OMP, origanum majorana L. polyphenols; OPA, orthophosphoric acid; Q, quercetin; RE, relative error; RMSD, root-mean-square-deviation; RSD, relative standard deviation; XP, extra precision.
